# AACVD system and protocol to fabricate CuO and Co_3_O_4_ nanostructured coatings for application as selective absorbent materials

**DOI:** 10.1016/j.mex.2023.102219

**Published:** 2023-05-12

**Authors:** D.A. Vázquez-Vargas, P. Pizá-Ruiz, P. Amézaga-Madrid

**Affiliations:** Departamento de Física de Materiales, Centro de Investigación en Materiales Avanzados, S.C. Chihuahua, Chih., C.P. 31136, México

**Keywords:** AACVD, Selective absorbent materials, CuO, Co_3_O_4_, AACVD system and protocol to fabricate CuO and Co_3_O_4_ nanostructured coatings for application as selective absorbent materials

## Abstract

In the present work, an aerosol-assisted CVD (AACVD) system is described, together with a representative example of the synthesis of nanostructured coatings, which is an attractive alternative to being implemented at the industrial level. The semi-automated AACVD system synthesizes thin films or coatings of nanostructured materials, mainly metal oxides, and noble metals. Its main components, as well as its operation, are presented here. This simple AACVD method makes it possible to produce the coatings at relatively low temperatures and in a single step. Finally, the synthesis of CuO and Co_3_O_4_ nanostructured coatings deposited on stainless steel substrates is reported, which are excellent candidates for use as selective absorbent materials. The CuO and Co_3_O_4_ coatings present high quality and purity; no further thermal treatments are required to obtain the pure and crystalline phases. The main highlights of the proposed method are as follows:

a) An AACVD System for depositing thin films and coatings designed and entirely fabricated at the Centro de Investigación en Materiales Avanzados, S.C.
b) A low temperature (350 °C) synthesis protocol to obtain CuO and Co_3_O_4_ nanostructured coatings on stainless steel substrates.
c) The CuO and Co_3_O_4_ coatings presented the optimum characteristics to be considered selective absorbent materials.

## Graphical abstract

**Figure:**
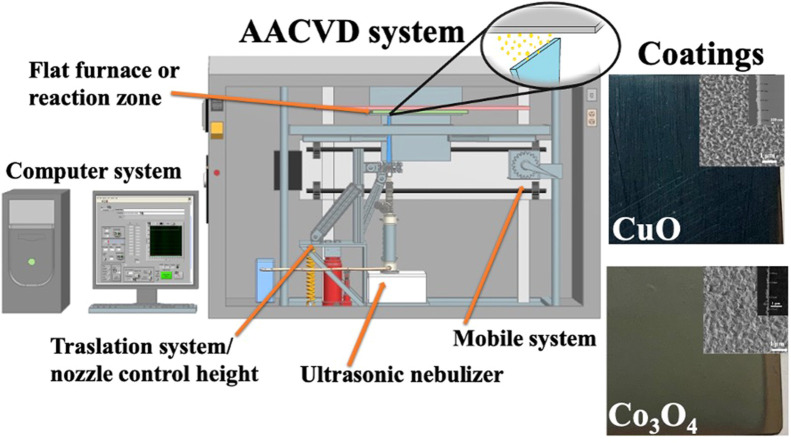

Specifications table

**Table:** 

Subject area:	Materials Science
More specific subject area:	Synthesis and characterization of coatings of metal oxides.
Name of your protocol:	AACVD system and protocol to fabricate CuO and Co_3_O_4_ nanostructured coatings for application as selective absorbent materials
Reagents/ tools:	
Experimental design:	The synthesis of coatings of CuO and Co_3_O_4_ on stainless steel by AACVD technique is described in detail; the system and essential parts are shown. The obtaining of these coatings by a simple methodology and relatively easy to install in laboratory is explained step by step.
Trial registration:	Not applicable
Ethics:	Not applicable
Value of the Protocol:	• An AACVD System for depositing thin films and coatings designed and entirely fabricated at the Centro de Investigación en Materiales Avanzados, S.C. The AACVD technique is simple, easy to manipulate, and inexpensive. • Materials are synthesized continuously in a single step, no subsequent heat treatments are required, no templates are needed to generate specific structures, no process control agents, and no buffer solutions are needed to achieve a specific pH. In addition, the materials are synthesized at relatively low temperatures. • The protocol to fabricate CuO and Co_3_O4 nanostructured coatings on stainless steel substrates at low temperature is presented in detail. The CuO and Co_3_O_4_ coatings presented the optimum characteristics to be considered as selective absorbent materials.

## Description of protocol

### Fundamentals of the AACVD method

Aerosol-assisted chemical vapor deposition (AACVD) is a widely explored method for synthesizing coatings [1], [2], [3], [4], [5], [6], [7], [8], [9], [10], [11], [12], [13], [14], [15], [16], [17] and nanostructured particles [10,[18], [19], [20], [21], [22], [23], [24], [25], [26]]. In this CVD variant technique, the reagents are dissolved in a precursor solution, which is transported to the reaction zone or heated substrate as a cloud of micrometric droplets through a carrier gas. A substrate is located on or near a heating plate to deposit thin films or coatings. The precursor solutions dilute organometallic or inorganic salts in some non-viscous solvents, such as alcohol, acetone, or water. Among other devices, an ultrasonic nebulizer can generate a droplet cloud or aerosols; it is an essential component of the AACVD systems, as it generates micrometric droplets of size depending on the ultrasonic frequency, surface tension, and density of the solution. The droplet size is typically 1 - 2 µm for 1 – 2 MHz of frequency and relatively diluted solutions. A carrier gas transports the aerosol to the reaction region, where its flow is optimized for each material. On their way to the reaction zone, the precursor's droplets transform physically and chemically to finally produce the material of interest. As the droplet's temperature increases, the solvent starts to evaporate; then, the solute could melt, sublimate, or decompose, followed by the diffusion of reactants and their chemical reaction to generate the nucleation and growth of the material. If the material is deposited on a substrate, other processes occur near the surface, such as the adsorption and surface diffusion of reactants, their chemical reaction, and the desorption and evacuation of reaction products [10]. There is a wide substrate temperature range for synthesizing binary components in which the desired materials are obtained with good uniformity, adhesion, smoothness, and homogeneous properties. Many types of materials and morphologies can be obtained, for instance, pure materials [[4], [5],[11], [12], [13], [14]], doped [2,9,15], composite [1], complex ternary materials [6], morphologies such as monolayer [5,[16], [17]] or multilayer coatings [7], nanorods [3], nanowires [4], or core-shell structures [3,8].

In the AACVD technique, the material is continuously obtained in a single step, without post-deposition annealing or other treatments, which is an important advantage because costs are reduced. In contrast, other techniques require additional heat treatment for a complete crystallization and stabilization of the microstructure. Additional advantages of this method are the relative simplicity, as it does not require sophisticated and expensive infrastructure, including at the industrial level, nor vacuum or pressure systems needed. The synthesis parameters, such as temperature, specific substrate selection, carrier gas flow, composition, and concentration of the precursor solution, must be optimized to produce high quality nanostructured materials, uniform, homogeneous, crack-free coatings, well adhered to the substrate surface, and chemically stable materials. The thickness of the material can also be controlled, obtaining materials in the form of coatings with thicknesses from a few nanometers to microns. This AACVD system can work with temperatures from 100 to 550 °C, it is an open system in the presence of atmospheric air, it is designed to synthesize metal oxides and some noble metals (Pt, Au, Pd, Ag).

In this work, we describe the AACVD system and present the synthesis of two metal oxide coatings: copper oxide (CuO) and cobalt oxide (Co_3_O_4_). These materials have very interesting and critical optical properties; a low band gap of around 1 to 3 eV, high absorption in the solar interval, and transparency to the near infrared, which make them excellent candidates to be applied, particularly as selective absorber materials. Complete and detailed information about the synthesis of these materials by this AACVD system, the materials, reagents, and equipment used, their microstructural characterization, optical properties analysis, band gap determination, and the selectivity of these two materials was published by Vázquez-Vargas et al. [5].

### Description of the experimental AACVD system to synthesize thin films

**Fig. 1:**
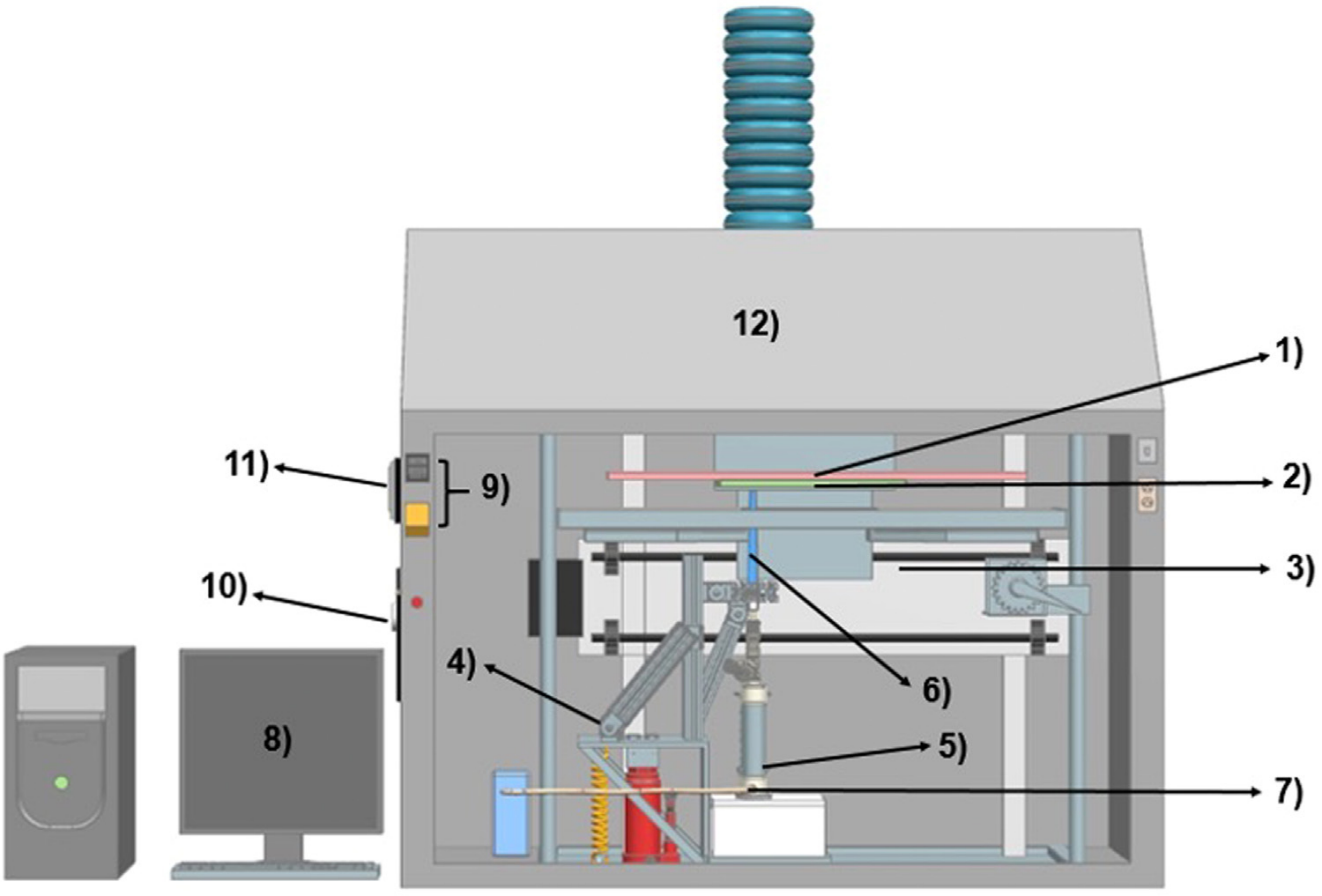
Schematic of the AACVD system for synthesizing thin films and coatings. Components: 1) flat furnace or reaction zone, 2) substrate holder incorporated to the 3) mobile system, 4) nozzle translation system; 5) ultrasonic nebulizer with accessories; 6) nozzle; 7) carrier gas inlet, 8) computing system, 9) temperature controller, 10) pressure controller, 11) flowmeter and 12) extraction hood.

Fig. 1 shows a schematic of the complete AACVD system, showing the main components and their interconnectedness. The system has 4 essential parts:

a) A reaction and heating zone, i.e., a flat furnace (1), which raises the substrate temperature to the selected synthesis temperature according to the material to be synthesized. The furnace reaches the programmed temperature by a set of electronic components, whose main elements are resistance and a thermocouple; in addition, to corroborate the furnace temperature, the system has a temperature controller (9) that allows keeping it constant during the whole deposition process.
b) A mobile system (3), where the substrate (2) is placed and is just 1 or 2 mm away from the furnace, thus allowing the heating of the substrate and also moving it from one side to the other as many times as programmed. The movement of the mobile system is controlled by the software located in the computer system (8). This program controls the movement of the substrate holder, the synthesis temperature, the flow of the carrier gas, and the number of cycles (or passes from one side to the other) to obtain the desired thickness.
c) An ultrasonic nebulizer (5), which transforms the precursor solution into a cloud of droplets or aerosol. Connected to the nebulizer chamber is a hose that allows the passage of the carried aerosol to the nozzle (6) and finally to the surface of the substrate. The carrier gas is regulated by a pressure controller (11) and a flow meter (10).
d) A translation system that allows the nebulizer and the nozzle (6) to be raised so that the nozzle-substrate distance is optimal for uniform deposition of the materials over the entire surface of the substrate. The AACVD system is located inside a fume hood (12) which serves as a safety device to prevent inhalation of fumes by personnel working with the system and to prevent contamination of the deposited material. Outside the fume hood on one side, the computer system is located. (8).

**Fig. 2:**
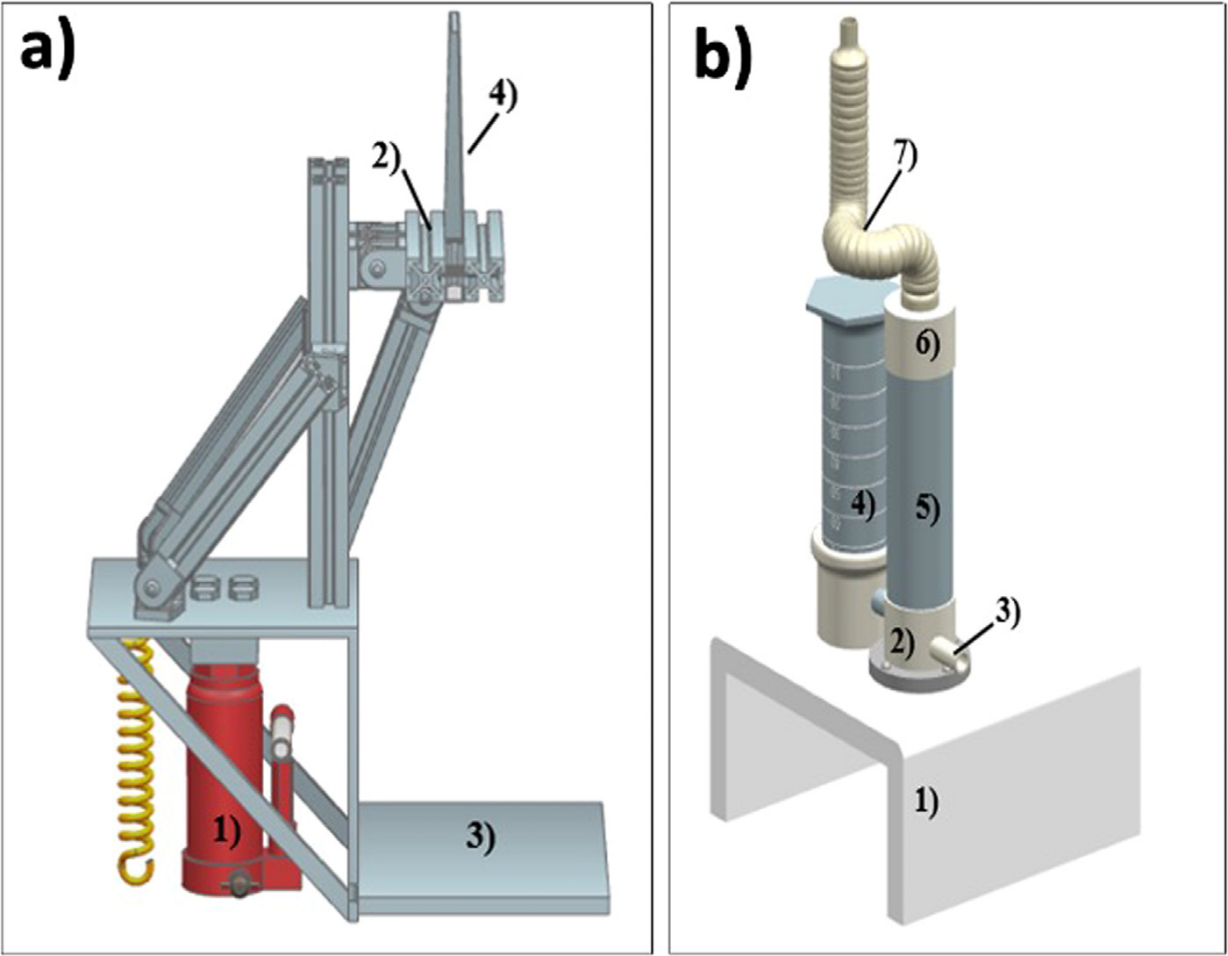
a) Translation system and its components: hydraulic jack (1), nozzle holder (2), nebulizer base (3), nozzle (4). b) Ultrasonic nebulizer and its accessories: base (1), nebulization chamber (2), accessories to connect the carrier gas (3), precursor solution container (4), droplet cloud container (5), plug (6) connecting the (5) with the hose (7) to be connected to the nozzle.

Fig. 2(a-b) shows two essential parts of the AACVD system in more detail. Fig. 2a presents the translation system's components, consisting of a base (3) where the ultrasonic nebulizer is placed close and vertically to the nozzle and is simply coupled. The hydraulic jack (1) raises the translation system with the nozzle to the surface of the furnace. On the other hand, the accessories of the ultrasonic nebulizer are presented in Fig. 2b; the nebulizer is made by an acrylic base (1) where a container for the precursor solution (4) and a nebulization chamber (2) are coupled, which has an electronic module with a ceramic piezoelectric that when turned on vibrates at a frequency that allows the transformation of the precursor solution to a cloud of droplets, the nebulization chamber also has an inlet for the carrier gas (3), which will transport the aerosol to the nozzle. To the container of the precursor solution, a test tube containing the precursor solution is coupled oppositely, thus allowing to generate of a vacuum that allows having a specific level in the mist chamber (5); adapted in the mist chamber are a container and a plug with a hole in the center (6) which will concentrate the aerosol, select the smallest drops and allow the transport by the gas through a hose (7) to reach the nozzle.

**Fig. 3:**
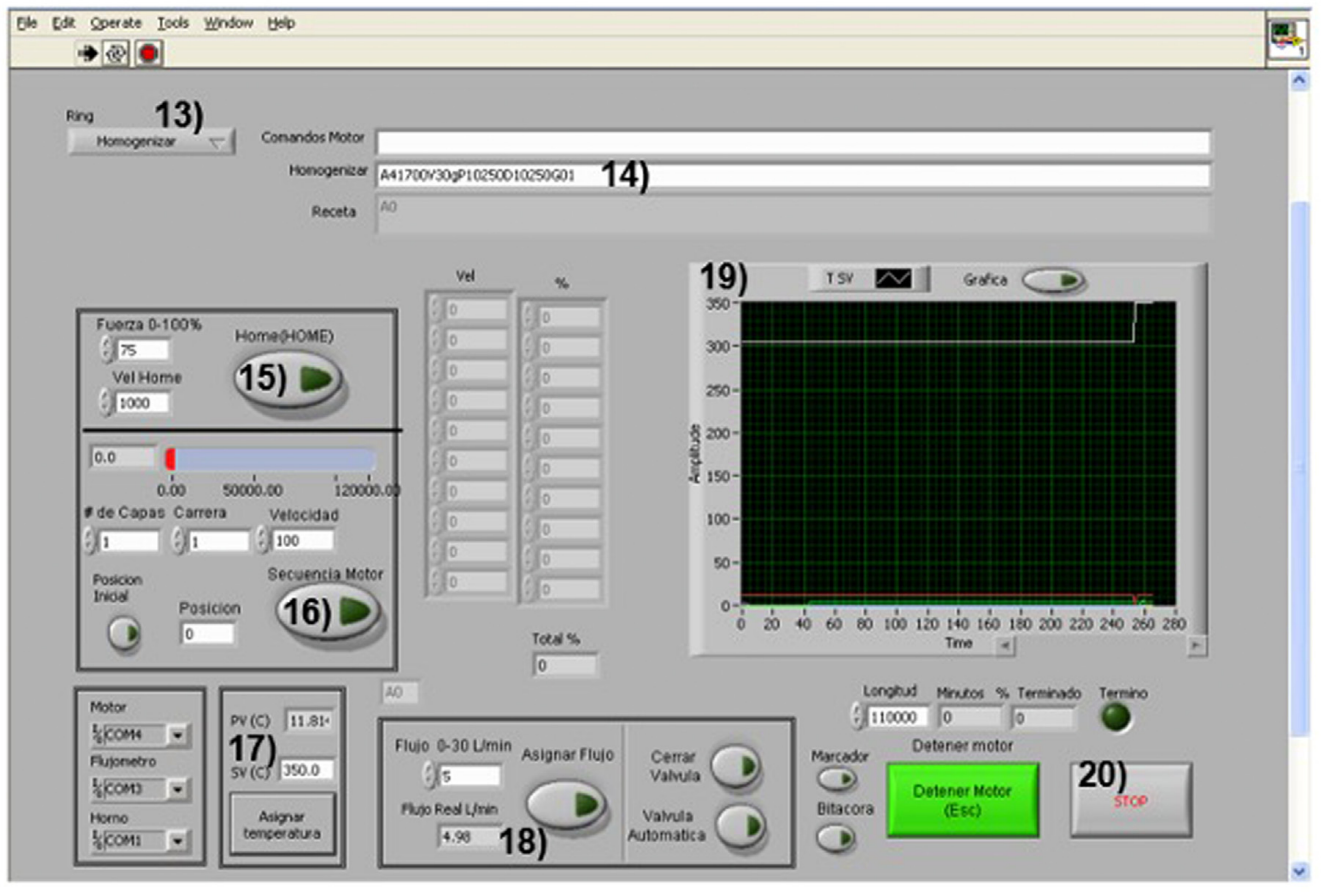
Illustration of the graphical interface. It allows the semi-automation of the AACVD system; in this interface, the necessary synthesis parameters are introduced to obtain the desired material.

Fig. 3 shows the synthesis program where the data of each synthesis parameter is captured for each material to be obtained. The software used was LabVIEW 7.1.

Steps to follow: a) Determine the type of program, set the substrate holder travel distances (13), b) set the start of the synthesis, the speed of the mobile system (in pulses per second because a stepper motor controls it), the nozzle-substrate distance and the number of cycles to determine the thickness (14). c) Indicates the start of each synthesis that guarantees that the system starts from the same starting point and the conditions or parameters already captured are carried out (15); d) button that allows starting the synthesis process, initiating the mobilization of the system (16); e) measures the temperature at which the furnace is located and the temperature at which it will be maintained in the synthesis process (17); f) establishes the flow of the carrier gas, monitors the actual flow, and finally (17); e) measures the temperature at which the furnace is and the temperature at which it will be maintained in the synthesis process (17); f) establishes the flow of the carrier gas, also monitors the actual flow, and finally (18), g) is the emergency button, which allows stopping the equipment in case the synthesis is not being carried out in the correct way (19).

### Materials and reagents necessary to synthesize CuO and Co_3_O_4_ coatings on stainless steel substrates

Table 1 shows the reagents needed for the synthesis of coatings.

**Table 1:** Materials and reagents used to synthesize CuO and Co_3_O_4_ coatings on stainless steel substrates.

Material/Reagent	Supplier	CAS
Stainless steel (2.5 × 2.5 cm)	–	–
Air gas	–	–
Methanol	J.T. Baker	67–56–1
Cobalt (II) acetate tetrahydrate	Merck	6147–53–1
Copper (II) nitrate hemi-pentahydrate	Merck	19,004–19–4

### Experimental procedure

**Fig. 4:**
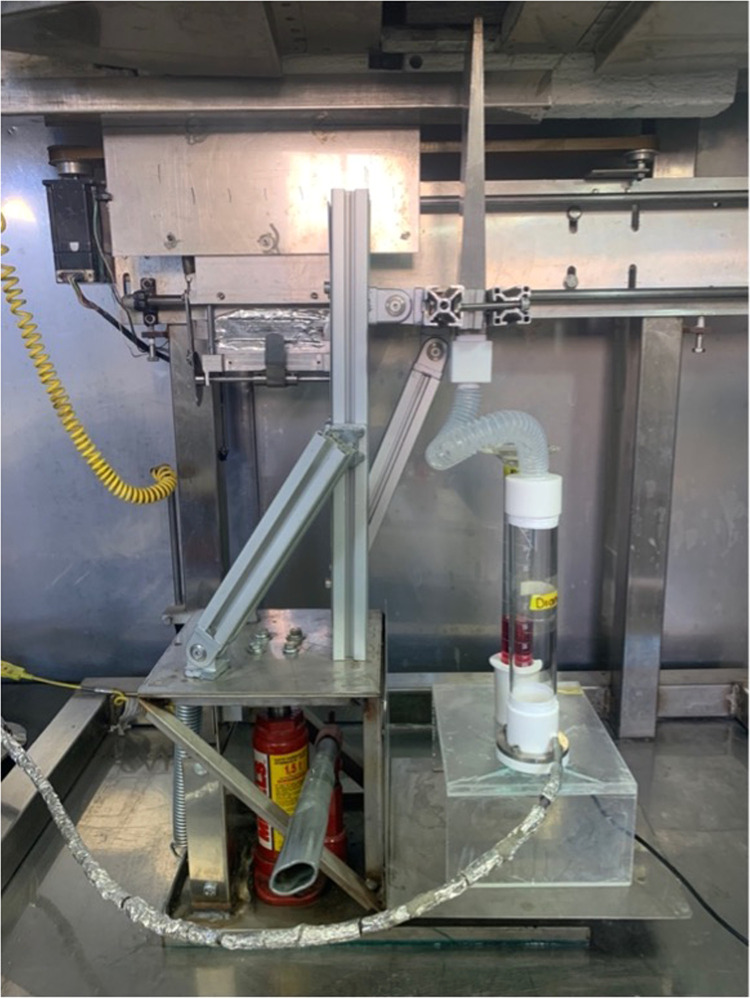
Actual photograph of the AACVD system parts: coupling the translation system with nozzle - nebulizer with its components and accessories.
d) Subsequently, the fume hood is turned on to stabilize the temperature in the whole system.
e) Then the substrate is placed and fastened to the substrate holder or heating plate; the deposition temperature is set, which in this case for the synthesis of the two coatings was 350 °C, and the substrate heating system is turned on.
f) Subsequently, the introduction of the carrier gas is started for its thermal stabilization, setting the flow rate for this case at 5 L min^−1^. The particular value of the carrier gas flow rate and the deposition temperature depends on the absorbent coating to be deposited and its characteristics.
g) Once the temperature of the substrate and the whole system (carrier gas, nozzle) has thermally stabilized, the precursor solution is introduced into the nebulizer. As supplementary material, video 1 is presented, showing how the precursor solution is poured into the nebulizer container (it is still outside the system to have a better appreciation of its components and accessories).
h) The nozzle displacement speed is set, in this case, 0.1 mm *sec*^−1^, a parameter that allows controlling the deposit characteristics, i.e. thickness and surface morphology.
i) The ultrasonic nebulizer is turned on to generate the aerosol cloud of the precursor solution.
j) Nozzle displacement is initiated once the action of the carrier gas stabilizes the aerosol flow. As the generated aerosol cloud approaches the upper end of the nozzle, the aerosol naturally heats up to a temperature below the deposition temperature. This preheating ensures that the precursor reaches the substrate surface in the deposition zone at the temperature required for thermal decomposition and proper surface diffusion, chemical reaction, nucleation, and coating growth occur. These physical-chemical transformations depend fundamentally on the substrate surface temperature, which is the determining parameter of the process.

a) The conventional methods and ultrasonic baths were used to clean the reflective metallic substrates. First, they are washed with water and phosphate-free soap to eliminate impurities or contaminants that affect the deposit of the absorbent coating; then, they are rinsed with water. Subsequently, the substrate was immersed in a beaker with acetone and placed in the ultrasonic bath for 20 min. After that time, the acetone is removed, and another wash is performed with methanol for the same interval. Once the sonication time is over, the methanol is removed, and the substrates are dried and stored to avoid contamination. In this case, a 2.5 × 2.5 cm stainless steel sheet was used as substrate.
b) The precursor solution containing the salts of the elements of interest, in this case, the Copper (II) nitrate hemi-pentahydrate salt, was used for CuO coatings at a molar concentration of 0.1 M in methanol; for Co_3_O_4_ coatings, the cobalt (II) acetate tetrahydrate salt was used at a molar concentration of 0.1 M in methanol.
c) The parts of the AA-CVD system are coupled: the nebulizer and the translation system with nozzle; the carrier gas is connected to the nebulizer, which is clean filtered air. Fig. 4 shows a photographic image of the nebulizer and translation system coupling of the AACVD system.

In the case of the chemical reaction process and the formation of both metal oxides, the chemical reactions involved in the formation process of each one is presented below: a) for obtaining Co_3_O_4_ and b) for obtaining CuO, are shown below:

$$a)\;3{\left(CH_{3}\text{COO}\right)}_{2}\text{Co}\cdot 4H_{2}O+CH_{3}\text{OH}+14O_{2_{\left(g\right)}}\;\Rightarrow {\text{Co}}_{3}O_{4}+23H_{2}O+13{\text{CO}}_{2\left(g\right)}$$

$$b)\;\text{Cu}{\left({\text{NO}}_{3}\right)}_{2}2.5H_{2}O+{\text{CH}}_{3}\text{OH}+O_{2_{\left(g\right)}}\Rightarrow \text{CuO}+4.5H_{2}O+{\text{CO}}_{2\left(g\right)}+2NO_{2\left(g\right)}$$

k) Residual gasses produced by the thermal decomposition of the precursor are continuously extracted through the fume hood to avoid contamination of the substrate surface and thus obtain high purity coatings.

As supplementary material, video 2 shows the AACVD system in operation. Once the synthesis time is over, the AACVD system and its parts are turned off. The fume hood is turned off, and we wait for the temperature to drop and for the coatings of the obtained materials to cool down.

**Fig. 5:**
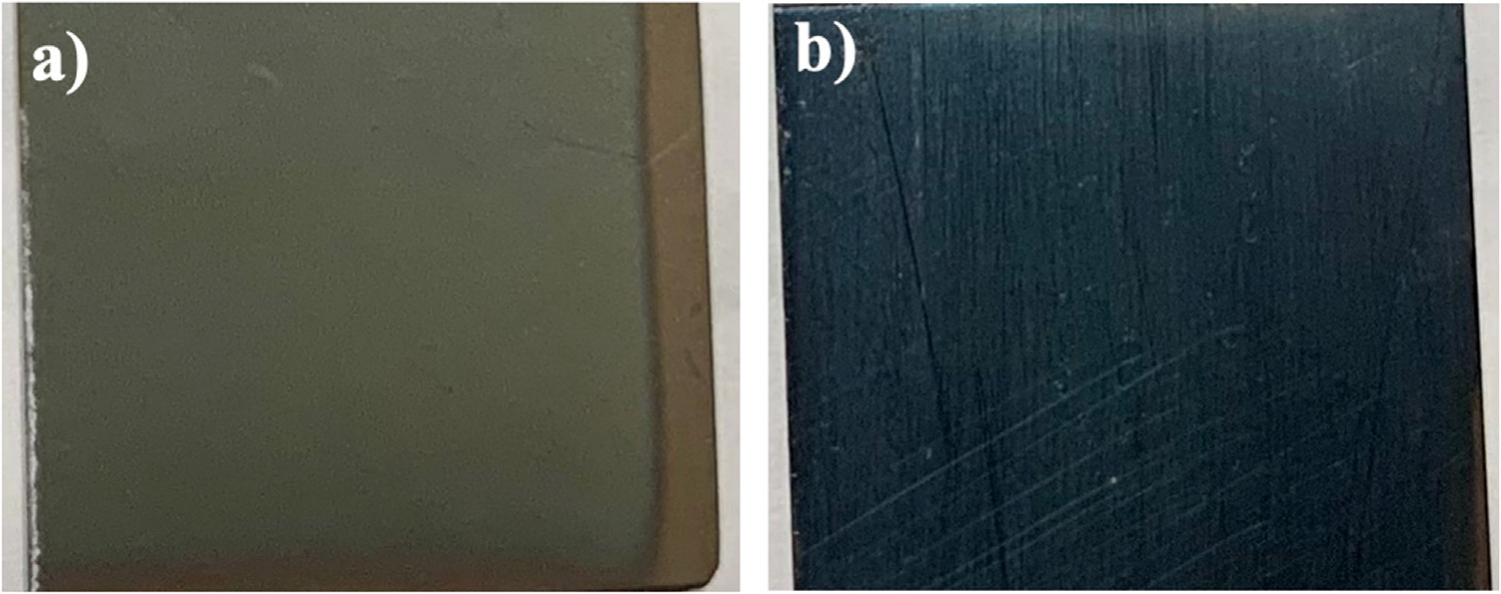
Coatings obtained by AACVD. a) Co_3_O_4_; b) CuO.

Fig. 5a and b show photographic images of the Co_3_O_4_ and CuO coating, respectively. Both were deposited onto stainless steel; at first glance, they present a dark coloration. In addition, homogeneous uniform layers free of cracks and firmly adhered to the substrate are visible. Important characteristics to consider as an optimal coating for the application as selective adsorbent material [5].

**Fig. 6:**
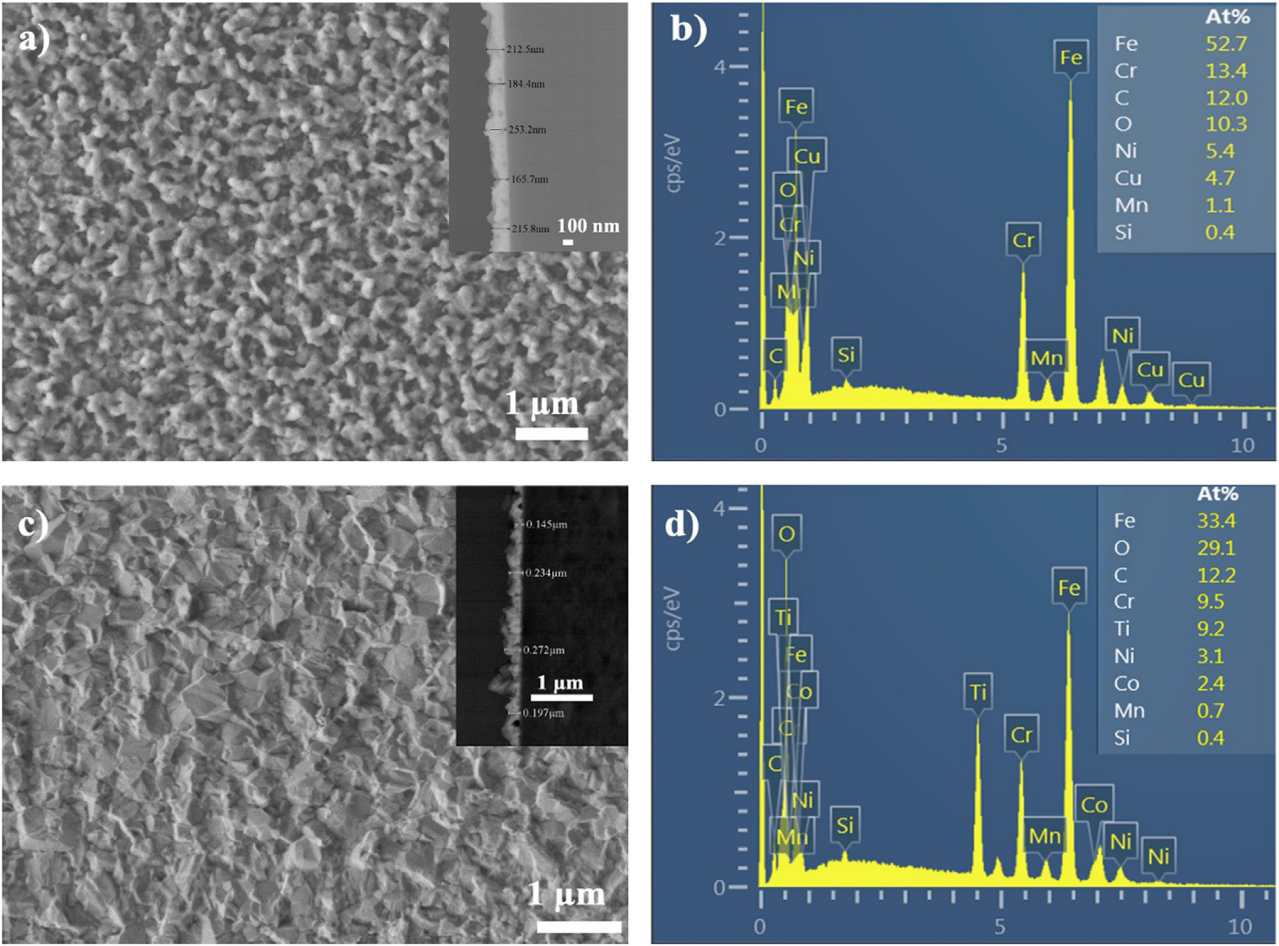
SEM micrographs and EDS analysis showing the material's surface morphology and elemental composition. a) and b) CuO on stainless steel. c and d) Co_3_O_4_ on stainless steel. The insets in a) and c) show the cross-section of the films, displaying their thickness.

Finally, Fig. 6(a-d) shows the morphology and elemental composition in the atomic percentage of the two materials analyzed by scanning electron microscopy. Fig. 6a shows the irregular, porous, dense, and rough morphology typical of CuO since when the synthesis of this material begins, the growth is in the form of agglomerates that form islands until the complete coating is formed [5]. The inset in Fig. 6a shows the cross-section of the nanostructures CuO coating, showing its thickness; a rough but homogeneous film with a thickness of approximately 204 nm on average can be observed. Fig. 6b shows the X-ray energy dispersive (EDS) analysis where the elemental composition in atomic percentage can be appreciated, the elements of interest, Cu and O, that form the coating, and the component elements from the substrate (stainless steel) are also presented. The presence of C is due to the solvent used to make the precursor solution.

On the other hand, Fig. 6c shows an SEM micrograph where the surface morphology of the Co_3_O_4_ coating displays a well-structured microstructure without pores, with irregular triangular grains. The inset in Fig. 6c presents the cross-section of the nanostructured Co_3_O_4_ coating showing an average irregular but homogeneous thickness of approximately 220 nm. Fig. 6d shows the EDS analysis showing the elemental composition in atomic percentage, the elements of interest Co and O that form the coating, and the component elements from the substrate (stainless steel) [5].

The microstructural and optical properties, thermal emissivity, solar absorptance determination, band gap determination, and selectivity of these materials are explained and discussed in detail by Vázquez-Vargas *et al*. [5], where it is concluded that CuO and Co_3_O_4_ coatings synthesized by this AACVD technique were suitable selective absorbing materials.

## Method validation

CuO and Co_3_O_4_ nanostructured coatings were deposited by AACVD, in a simple one-step manner, with high purity and quality, well adhered to the substrate, with uniform and homogeneous characteristics on stainless steel metal substrates. The experience in working with this AACVD system and method has been more than a decade [1], [2], [3], [4], [5], [6], [7], [8], [9], [10]. It is a reproducible method, repeatable as often as necessary, and the metal oxide materials of interest are obtained as nanostructured coatings. The selective absorber coatings were correctly deposited, with the appropriate thickness and microstructure for the application of interest, and also confer to the surface of the reflecting metal substrate new optical properties, a darker coloration that leads mainly to a high absorptivity in the solar range. This coating, together with the infrared reflective property of the metallic substrate, will form a selective absorber plate applicable for the possible economical fabrication of photothermal solar collectors in the near future.

## CRediT authorship contribution statement

**D.A. Vázquez-Vargas:** Methodology. **P. Pizá-Ruiz:** Methodology. **P. Amézaga-Madrid:** Conceptualization, Methodology, Writing – review & editing.

## Declaration of Competing Interest

The authors declare that they have no known competing financial interests or personal relationships that could have appeared to influence the work reported in this paper.

## Data Availability

No data was used for the research described in the article.

## Appendix Supplementary materials

Image, application 1

Image, application 2
